# Use of Frogs as a Model to Study the Etiology of HLHS

**DOI:** 10.3390/jcdd10020051

**Published:** 2023-01-29

**Authors:** Shuyi Nie

**Affiliations:** School of Biological Sciences, Petit Institute for Bioengineering and Bioscience, Georgia Institute of Technology, Atlanta, GA 30332, USA; shuyi.nie@biology.gatech.edu

**Keywords:** frog, congenital heart disease, hypoplastic left heart syndrome

## Abstract

A frog is a classical model organism used to uncover processes and regulations of early vertebrate development, including heart development. Recently, we showed that a frog also represents a useful model to study a rare human congenital heart disease, hypoplastic left heart syndrome. In this review, we first summarized the cellular events and molecular regulations of vertebrate heart development, and the benefit of using a frog model to study congenital heart diseases. Next, we described the challenges in elucidating the etiology of hypoplastic left heart syndrome and discussed how a frog model may contribute to our understanding of the molecular and cellular bases of the disease. We concluded that a frog model offers its unique advantage in uncovering the cellular mechanisms of hypoplastic left heart syndrome; however, combining multiple model organisms, including frogs, is needed to gain a comprehensive understanding of the disease.

## 1. Highly Conserved Heart Development in Vertebrates

Heart development in vertebrates is highly conserved, allowing us to use animal models such as mice and frogs to gain important insights into human heart development. Briefly, two bilateral pools of cardiac progenitors are specified in the mesoderm next to the organizer (or node depending on species), they next migrate ventrally to meet at the ventral midline. Fusion of the two cardiac primordia is followed by the formation of the primary heart tube, with a myocardial layer enclosing the inner endocardial tube. Next, the linear heart tube undergoes a morphogenesis event, including looping and myocardium expansion (ballooning). Shortly after this, the heart starts to beat due to contractility of the cardiomyocytes. Subsequently, the heart compartmentalizes to make different chambers through septation and valve formation. Heart chambers also undergo a further morphogenesis process to adopt distinct morphological features, including the trabeculation of the ventricular wall. While the frog has a three-chambered heart with only one ventricle, this single ventricle is believed to resemble the mammalian left ventricle based on its tissue of origin [1], making it more feasible to study single ventricular diseases such hypoplastic left heart syndrome.

In addition to similarities at the cellular level, heart development in different vertebrate animals is regulated by a common cohort of regulators, such as Nkx2.5, Tbx5, Islet1 [2,3,4,5]. The evolutionally conserved regulators for heart development have been summarized in several reviews [6,7]. At the genomic level, sequence annotation and assembly of Xenopus tropicalis has demonstrated high levels of gene synteny and orthology between the frog and human genome [8,9,10]. At the protein level, cardiac proteomes of four vertebrate models have been compared and conserved proteins and pathways have been identified. Species-specific protein enrichment has also been uncovered, with certain proteins known to play critical roles in heart development and disease (e.g., Notch1) enriched in the frog heart, but not in the hearts of the mouse or pig [11]. These results further demonstrate the suitability of using a frog model to study human heart diseases.

## 2. The Frog Is a Powerful Model for Studying Congenital Heart Diseases

Besides the above similarities in vertebrate heart development, the frog also offers the unique advantage in studying heart development and disease. Frog embryos develop externally, so it is easy to examine heart development at different stages and follow the pathological progression of heart defects. Moreover, the frog embryos can survive without a functional heart; thus, cardiovascular defects that are otherwise embryonic-lethal can be examined for an extended period of time in frog embryos. A frog can breed year-round and has a large litter size (>1000 eggs per frog per day). Through in vitro fertilization, large numbers of synchronized embryos can be obtained. Combined with rapid development of frog embryos (it takes around 3 days to reach the chamber heart stage), experiments can be performed quickly with sufficient sample size and controls performed in parallel. On top of these convenient features of frog embryos, lots of tools and resources have been generated for studying the functions of genes during frog heart development. First, a well-defined fate map of early frog development has been described [12,13] so that precise targeting can be achieved. For example, the left or right side of the embryo can be targeted at the 2-cell stage by injecting into one of the blastomeres, leaving the contralateral side as an internal control. To target the cardiac tissue, dorsal lateral marginal zone cells can be injected at the 8-cell to 32-cell stages. Functional studies of single or multiple genes can be easily achieved by simple gain-of-function or loss-of-function approaches. If certain genes play additional roles in other tissue, they can be specifically targeted to prospective cardiac tissue at the cleavage stages to avoid the interference from additional defects. The large size of a frog embryo makes this type of microinjection feasible. In addition, the exceptional healing ability of frog embryos allows for surgical manipulations such as transplantation to be used in combination with gain- and loss-of-function approaches to further dissect tissue requirements of the genes. For example, to determine whether a gene acts in a cell-autonomous manner in heart mesoderm, heart field tissue can be dissected from an embryo with this gene knocked down and inserted into a wild type host embryo. In parallel, a transplantation of a wild type heart field donor into a mutant host can be generated as a control. Heart field tissue is a small rectangular-shaped tissue just posterior to the developing cement gland, and can be easily dissected and transplanted at early tailbud stages (stage 22–26) [14,15,16]. Simply dissected, heart field and other tissue from frog embryos can also be cultured easily in a simple saline medium, thanks to the partitioning of the yolk in embryonic cells. Besides heart field tissue, the naïve animal cap cells of frog embryos can be induced to become beating heart cells by treatment with growth factors such as Activin [17,18,19,20], so that the activity of different cardiac genes can also be tested in this simple in vitro system. In vivo, due to the sub-superficial position of the heart and the partial transparency of later-stage tadpoles, the heart can be imaged directly in live animals to assess functional defects in addition to structural defects [21]. While a frog is not typically viewed as a genetic model system, with the Sleeping Beauty transposases and new tools of genome editing (CRISPR/Cas system) and the complete sequencing of the *X. laevis* and *X. tropicalis* genomes [9,22], it has also become a member of the genetic model organism. A number of transgenic frog lines are already available from the Xenopus resource centers (https://www.mbl.edu/research/resources-research-facilities/national-xenopus-resource, https://xenopusresource.org/ (accessed on 26 January 2023)), including lineage tracer lines (such as Myl3:GFP that labels cardiomyocytes [23] and Kdr:GFP that labels endothelial/endocardial cells [24]) and knockout mutant lines. In addition, mosaic F0 transgenic animals can be used for rapid analysis of phenotypes just days after injection and has been used successfully in the investigation of many genes during development [25,26,27,28,29,30]. 

With the tools described above, gene functions in frog heart development can be quickly analyzed. In fact, studies done with frogs have contributed to much of the early knowledge of vertebrate heart development [31,32,33,34,35,36] and has led to significant advancements in our understanding of human heart development and congenital heart diseases (reviewed in [37,38,39]). For example, the frog model has been used in the investigation of the role of Nkx2.5 and GATA4 in atrial septal defects [40,41,42], Tbx1 in DiGeorge syndrome [23,43], Tbx5 in Holt-Oram syndrome [44,45,46], Zic3 and Notch in heterotaxy [47,48,49,50,51,52,53], Chd7 in CHARGE syndrome [54,55], and Ets1 in Jacobsen syndrome [56]. 

## 3. Hypoplastic Left Heart Syndrome

Hypoplastic left heart syndrome (HLHS) is one of the most severe congenital heart diseases and accounts for nearly 25% of cardiac deaths in the first year of life [57]. This is because the left ventricle that pumps blood to the systemic circulation including coronary circulation is malformed and largely non-functional in HLHS. Besides the underdevelopment of the left ventricle, additional characteristics of HLHS include defects in the mitral valve and aortic valve (mitral/aortic stenosis or atresia). Babies born with HLHS require multiple palliative surgeries to redirect the right ventricle for systemic circulation and ultimately require a cardiac transplant for survival. Despite the severity of the disease, we still do not have a clear understanding of the cause of HLHS. Two major challenges in elucidating the mechanisms of HLHS are the multifactorial nature of the syndrome and the lack of relevant animal models that faithfully describe the disease. Multiple genes have been reported in association with HLHS, including Nkx2.5 [58,59], Notch1 [60,61,62], Hand1 [59,63], rbFOX2 [64], and Ets1 [65]. However, specific genetic causes are only known in a small subset of patients [66]. Moreover, significant anatomical variability is observed among different HLHS patients, suggesting that multiple animal models may be necessary to represent different anatomical subtypes of HLHS. This phenotypic variability makes the elucidation of the molecular and cellular mechanisms underlying the syndrome even more difficult. For a comprehensive review of HLHS, see [67,68].

## 4. The Frog as a Model for HLHS

Based on the advantages of the frog model described above, the frog model can contribute to the research of cellular processes and molecular pathways involved in the abnormal cardiac development in HLHS. A frog represents an efficient model to screen for causal factors of HLHS. Combining rapid transgenics and histological, molecular, and live imaging approaches, we can quickly determine if mutating one or several genes results in HLHS-like phenotypes in frogs. Such information will contribute to the establishment of animal models for HLHS, allowing for subsequent analysis for the molecular and cellular mechanisms during the pathological progression of HLHS.

The etiology of HLHS is likely manifold. One possibility is defects in the cardiac neural crest cells, which may result in stenosis or atresia of the aortic valve and abnormal configuration of the great arteries that are often associated with HLHS. Such defects may affect the outgrowth of the ventricle by impairing blood circulation, as suggested by the “no flow no growth” theory [69]. Several potential causal genes of HLHS have been identified along this line, including Connexin 43 (Cx43) [70]. Loss of Cx43 in mice resulted in outflow tract defects [71,72,73,74], indicating a role of Cx43 in cardiac neural crest development. However, how Cx43 regulates cardiac neural crest cells has been unknown until recently, when Cx43 was studied in frogs. Kotini et al. was able to show in frogs that Cx43 directly regulates N-cadherin expression to control the migration of cranial and cardiac neural crest cells. They dissected the functional domains of Cx43 and found that the carboxy tail of Cx43 is translocated into the nucleus, where it forms a complex with RNA Pol II. This complex directly binds to the promotor of N-cadherin to regulate N-cadherin transcription [75]. This study also mechanistically connects Cx43 with another potential causal factor of HLHS, N-Cadherin [76]. Similarly, genetic analysis of patients with non-syndromic left ventricular outflow tract obstruction (obstruction of LVOT, a broader spectrum of cardiac defects including HLHS) vs HLHS and coarctation of the aorta identified MCTP2 as another potential factor for HLHS. Since only one allele of functional MCTP2 is lost in the syndromic patient, the authors used a frog to perform carefully titrated knockdown analysis and found that partial loss of MCTP2 led to outflow tract defects including the loss of endocardial cushions therein [77]. This defect likely results from a failure in endocardial–mesenchymal transition, which is also regulated by another HLHS-related gene Notch1 [78]. 

Another possibility for the pathogenesis of HLHS is defective development of cardiomyocytes, which can lead to failure in ventricular outgrowth. Multiple genes have been reported to regulate cardiomyocyte development, from their early specification at cardiac crescent stage, to their proliferation, differentiation, and morphogenesis at the heart tube and chamber stages. While many conserved cardiogenic genes described at the beginning of this article play fundamental roles in heart development and are thus less likely to be directly involved in the etiology of HLHS, factors such as Islet1 (Isl1) and Notch1 can modulate the balance of different lineages of cardiac cells to control the size of cardiomyocyte population. For example, Notch1 activation suppresses cardiomyocytes while Isl1 suppresses the ventricular lineage of cardiomyocytes [79,80]. After early specification, proliferation of cardiomyocytes is carefully regulated, resulting in cardiac outgrowth and morphogenesis. Focal adhesion kinase (FAK) is a factor reported to regulate this process of heart development [81]. When FAK was knocked down in the frog, cardiac specification was not affected (reflected by normal expression of Nkx2.5, Tbx5, Tbx20, tropomyosin, and Troponin T). However, cardiac looping was significantly impaired. Since cardiac looping results from a different rate of cell proliferation in the left vs right side of the linear heart tube, the authors examined cell proliferation and found that FAK knockdown led to a significant decrease in the cell proliferation rate. They further demonstrated that FAK acts downstream of the FGF pathway to regulate cardiomyocyte proliferation and cardiac outgrowth [81]. Cardiac morphogenesis also depends on cell–cell adhesions. Yamagishi et al. reported that tight junction protein Claudin5 plays an important role in cardiac outgrowth in frogs. When Claudin5 was knocked down, cardiomyocytes failed to organize into tissue layers and cardiac outgrowth was severely affected [82]. Since tight junctions are linked to actin cytoskeleton, the author also found that the loss of Claudin5 disrupted the organization of sarcomeres. Two additional reports focused on the important role of actin filaments in cardiomyocyte differentiation and ventricular morphogenesis. In one report, a CapZ interacting protein Rcsd1 was shown to mediate non-canonical Wnt signaling to regulate the differentiation of cardiomyocytes. Loss of Rcsd1 in the frog led to a severely underdeveloped heart similar to that of HLHS with a slit-like ventricle [83]. In the other report, an ADP-ribosylhydrolase protein Adprhl1 was investigated in frog heart development. This protein is selectively expressed in ventricular cardiomyocytes and loss-of-function experiments led to disrupted myofibril assembly and strong defects in ventricular outgrowth and performance [84]. The authors followed up later with CRISPR-mediated transgenics to confirm the functions of Adprhl1 in the myofibril assembly and ventricular morphogenesis [85]. 

**Figure 1:**
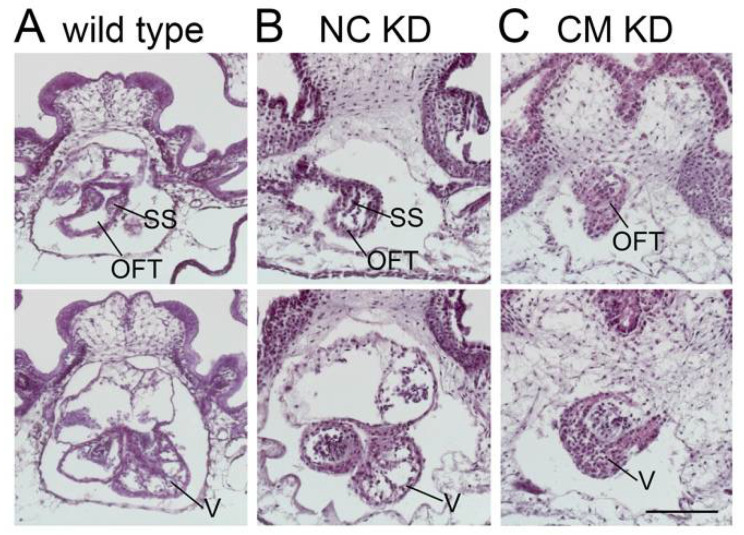
Ets1 knockdown in the cardiogenic mesoderm leads to HLHS-like phenotype in the frog. Transverse sections through (**A**) wildtype, (**B**) neural crest knockdown, and (**C**) cardiac mesoderm knockdown embryos are shown. Ets1 knockdown in the neural crest cells results in outflow tract defects but does not affect ventricular development significantly. In contrast, Ets1 knockdown in the cardiac mesoderm results in an underdeveloped ventricle with reduced chamber volume, mimicking the HLHS phenotype. SS, siral septum; OFT, outflow tract; V, ventricle. Scale bar = 200 um. (Reproduced with permission from [56].).

A third possible mechanism for the development of HLHS is defective development of endocardial/endothelial cells. The endocardial/endothelial defects may subsequently affect the proliferation of cardiomyocytes. Multiple genes have been implicated in frog endocardial/endothelial development, including Ets1, Ets1-related transcription factor Erg, and transcription factors Smad3 and GATA4 [86,87,88]. Among these transcription factors, Ets1 is considered as a causal factor of HLHS. The deletion of the Ets1 locus is the genetic cause for Jacobsen syndrome, in which HLHS is over-represented [89,90]. However, Ets1 KO in mice results in ventricular septal defects, double outlet right ventricle, or ventricular non-compaction, depending on the tissue where Ets1 is deleted, but not HLHS [91,92,93]. We have examined the role of Ets1 in frog heart development. When Ets1 was knocked down in the frog, we observed an HLHS-like phenotype [56]. Based on the well-defined fate map of the frog embryos, we were able to further dissect the function of Ets1 in different tissue. When Ets1 was knocked down in the cranial and cardiac neural crest cells, neural crest delamination and migration was defective, which resulted in defective aortic arch arteries made by cardiac neural crest cells. When Ets1 was knocked down in cardiogenic mesoderm, the ventricular development was impaired. The ventricle is stacked with piles of unorganized cells with significantly reduced ventricular chamber size and lack of trabeculation (Figure 1). This phenotype is due to the loss of Ets1 in mesoderm alone since transplantation of wild type heart field tissue into an Ets1 morphant embryo can successfully rescue the ventricular defects (unpublished). Using advanced light field microscopy, we were able to analyze heart performance in live tadpoles and confirmed that these smaller ventricles in mutant tadpoles also suffer from significantly reduced contractility (unpublished results). RNA sequencing analysis further confirmed that many genes involved in actomyosin contractility were down-regulated upon Ets1 knockdown (unpublished). The available transgenic animals such as Myl3:GFP (labels cardiomyocytes [23]) and Kdr:GFP (labels endocardial/endothelial cells [24]) can be used to further determine the role of Ets1 in the development of different cardiac cell populations. Using iPSCs derived from HLHS patients, Miao et al. has demonstrated intrinsic endocardial defects including abnormal endocardial-to-mesenchymal transition and extracellular matrix organization [94]. Coculture of these defective endocardial cells with cardiomyocytes led to impaired proliferation and maturation of cardiomyocytes. The functional crosstalk between endocardial and myocardial cells depend on fibronectin signaling [95,96], and defects in fibronectin signaling is also observed in our Ets1 mutant frog heart. This result further supports a role for endocardial cells in a syndrome where myocardial development is defective. Investigations of signaling pathways involved in endocardial/endothelial development can help us further understand the critical role of endocardial/endothelial development in the pathogenesis of HLHS. 

Despite recent advances made, there are clearly challenges in using frogs to model HLHS. The major ones are the different contribution of cardiac neural crest cells in heart development and the lack of right ventricles to investigate the different physiological features of the two ventricles. Since cardiac neural crest cells contribute to the development of great arteries including aorta, defects in cardiac neural crest migration and differentiation likely results in aortic stenosis/atresia that often associates with HLHS. However, in mammals, cardiac neural crest cells also migrate into the outflow tract and the proximal portion of the ventricle and contribute to the development of the septum there. Such a role is absent in a frog; therefore, preventing a complete analysis of cardiac neural crest cells in the development of HLHS. In mammals, cardiomyocytes in the left and right ventricle come from different sources. Cardiomyocytes in the left ventricle mainly come from the first heart field while cardiomyocytes in the right ventricle mainly come from the second heart field. Such a difference of origin may lead to different features of the two ventricles, including their tissue organization and contractility. These differences may account for the failure of the right ventricle after palliative surgeries in HLHS patients, when a right ventricle is redirected to function as a left ventricle. Since a frog only has one ventricle and its tissue origin is similar to that of the left ventricle in mammals [1], it cannot be used to study the physiological differences between the two ventricles. 

Despite potential differences between the ventricles, the single ventricle of a frog heart may offer unique opportunities to explore potential solutions for single ventricular diseases. With a single ventricle, the frog heart developed a strategy to partially separate systemic and pulmonary circulation. The frog heart has two atriums, so that the deoxygenated blood from systemic circulation and the oxygenated blood from pulmonary circulation are fully separated when returning to the heart. They are then be pumped into the same ventricle and back to circulation through a single outflow tract that has been divided by a spiral septum. To understand the unique anatomy and physiology of the frog heart, a study was performed recently using echocardiography and cardiac magnetic resonance imaging [97]. Their work suggested that a spongiform ventricular and the positioning of the atrio-ventricular valves and truncal valve likely prevents complete mixing of oxygenated and deoxygenated blood. For example, the deoxygenated blood seems to drain through the right atrio-ventricular valve into the superior and anterior part of the ventricle before exiting through the semilunar truncal valve into the partially septate outflow tract. Further investigation of the flow dynamics in the frog heart and great arteries will help us understand how the anatomical configuration of a single ventricle leads to functionally divided systemic and pulmonary circulations. Such knowledge may shed light on the therapeutic solutions in treating HLHS hearts. 

## 5. Conclusions

The frog represents an important model for studying congenital heart diseases, including HLHS. Studies using frogs have shed light on several cellular processes in heart development that may contribute to the pathogenesis of HLHS. However, every model organism has its limitations. When studying complex diseases such as HLHS, it is beneficial to use more than one model organism, to take advantage of the merit of each to gain a comprehensive understanding of disease etiology.

## Data Availability

Not applicable.
